# Effect of gender-affirming treatments on depression and anxiety symptoms in transgender people: a retrospective cohort study

**DOI:** 10.3389/fpsyt.2025.1709778

**Published:** 2026-01-05

**Authors:** Chun Yip Wong, Sze Ting Joanna Ngan, Pak Wing Calvin Cheng, Wing Ki Tang, Lai Yin Chow, Wai Kwok Kam

**Affiliations:** 1 Department of Psychiatry, Hospital Authority, Hong Kong, Hong Kong SAR, China; 2 Department of Psychiatry, The University of Hong Kong, Hong Kong, Hong Kong SAR, China; 3 Gender Identity Clinic, Department of Psychiatry, Prince of Wales Hospital, Hong Kong, Hong Kong SAR, China; 4 Asian Academy of Family Therapy, Hong Kong, Hong Kong SAR, China

**Keywords:** depression, gender congruence, gender-affirming hormone therapy, gender-affirming treatments, transgender

## Abstract

**Introduction:**

Given the unique mental health challenges among transgender individuals, it is imperative to understand the effectiveness of gender-affirming treatments in alleviating psychological distress. The existing literature gap calls for studies with larger sample sizes, extended follow-up periods, and rigorous controls for confounding variables. This study aims to explore if gender-affirming surgery (GAS) and gender-affirming hormone therapy (GAHT) is associated with improvement in symptoms of depression, anxiety, and gender incongruence among transgender individuals attending a specialist clinic in Hong Kong.

**Methods:**

In this retrospective cohort which consisted of 394 treatment-seeking transgender participants at baseline, 178 individuals were recruited from October 2023 to June 2024 and completed self-rated questionnaires to assess depressive and anxiety symptoms and gender incongruence. Socio-clinical profiles and questionnaire scores were compared among treatment status groups. Longitudinal changes in mental health outcome scores were analyzed using linear mixed-effects regression models, incorporating time-by-group interactions and random intercepts to account for individual baseline differences.

**Results:**

Gender-affirming treatments did not reduce depressive or anxiety symptoms significantly, after controlling for coping and social support. However, both GAS and GAHT were associated with significant improvements in gender congruence over time. Mood symptoms were significantly associated with social support and coping. Various sociodemographic and psychosocial factors, including employment status, living situation, psychological care, and psychiatric medication use, were associated with psychological outcomes.

**Discussion:**

Gender-affirming medical treatments alone may not entirely resolve the mental health difficulties faced by transgender individuals. Future research needs to better elucidate the impacts of persistent psychosocial challenges such as discrimination and rejection, as well as potential treatment complications. The treatment-seeking nature of this cohort, coupled with baseline psychiatric interventions, may have obscured significant correlations. While gender-affirming treatments can enhance gender congruence, our findings highlight the importance of holistic approaches to strengthen adaptive buffering mechanisms throughout the gender transition process.

## 1 Introduction

The term “transgender” can refer to individuals whose gender identity differs from their sex assigned at birth (1). However, these terms can also be ambiguous, sometimes encompassing only those who identify as girls/women or boys/men, or including those who are gender nonconforming. Transgender and gender diverse (TGD) serves as an umbrella term that includes transgender and nonbinary individuals, as well as those who have engaged with a gender clinic or received a gender-related diagnosis, such as “gender identity disorder”, “gender dysphoria”, or “gender incongruence” (1). Historically, the DSM-IV (2) classified such experiences under “Gender Identity Disorder” (GID), defined by distress arising from a mismatch between biological sex and psychological gender. However, growing consensus recognizes that gender incongruence itself is not a mental disorder, but rather that distress may result from this misalignment. Accordingly, the DSM-5 replaced GID with “Gender Dysphoria” (3), and the ICD-11 reclassified “Gender Incongruence” under sexual health, thereby depathologizing gender diversity while preserving access to medical care (4). For consistency, this thesis will employ the term “gender incongruence” to denote the condition also referred to as gender dysphoria.

A substantial body of empirical research has elucidated the disproportionate mental health difficulties within the TGD population, documenting significantly higher rates of depressive symptoms (5, 6), anxiety (7), and psychiatric comorbidities (8, 9) relative to cisgender controls. This elevated risk has been consistently observed across studies, as concluded by recent publications of systematic reviews and meta-analyses (10, 11). The 2019/20 Hong Kong Transgender Survey, the most comprehensive community-wide online survey to date, revealed that approximately one-third of respondents reported moderate-to-severe levels of depressive and anxiety symptoms (12).

Several theories have been proposed to explain this heightened distress. These include the inherent gender incongruence, diminished social support, limited access to healthcare services, and minority stressors (13, 14). Having a gender identity unaligned with one’s sex assigned at birth may cause clinical distress and impairment in functioning. Furthermore, the gender minority stress model (15, 16) posits that TGD people face unique stressors (e.g. discrimination, harassment, abuse and internalized anti-trans attitudes) that contribute to chronic marginalization and isolation, eventually causing adverse psychological outcomes.

TGD individuals often seek gender-affirming care, which the World Professional Association for Transgender Health (WPATH) recommends tailoring to each person’s needs. This may include psychological support, gender-affirming hormone therapy (GAHT), and gender-affirming surgeries (GAS) (17). Evidence from systematic reviews suggests that GAHT can improve depressive symptoms (18, 19), quality of life (20, 21), body satisfaction (22), self-esteem (23), and interpersonal functioning (24). It may also enhance community connectedness (25) and mitigate minority stress through increased healthcare access (26, 27). However, methodological shortcomings limit the generalizability of findings. Issues such as reliance on group means, small predominantly Western samples, short follow-up periods, and failure to control for social support or coping strategies (18, 28–31). Additionally, since the full psychological effects of GAHT may take up to five years to develop, most existing studies may underestimate its long-term impact.

Following hormone therapy, many individuals consider gender-affirming surgeries (GAS) as a crucial step in their transition. Research on GAS shows similarly mixed outcomes. While some studies report improved quality of life (32, 33), sexual health (34), self-esteem (35), and gender congruence (36, 37), systematic reviews reveal inconsistent effects on mood symptoms. For example, Shelemy et al. (38) found that only 3 out of 8 prospective studies showed reductions in depression, with no changes in anxiety across 4 studies (38). These discrepancies may be attributed to persistent limitations, including small sample sizes, lack of adjustment for confounding variables such as psychiatric comorbidity, sociodemographic background, and concurrent GAHT (37, 39), underscoring the ongoing need for more rigorous and representative research. Our current study aimed to fill these research gaps by including a larger cohort of treatment-seeking TGD participants to assess the impacts of gender-affirming treatments on mental health outcomes.

Social support and coping are recognized as key determinants of mental health (40, 41). Social support, defined as the perception that help will be available when needed (42), has been shown among TGD populations to reduce risks of depression (43, 44), anxiety (45), self-injury (8, 46), and suicidality (47). It further buffers the negative effects of gender minority stress (48–50). However, levels of social support vary across cultures; for example, Asian TGD individuals generally report lower support than those in Western societies (51, 52), suggesting that TGD people in Hong Kong may face particularly limited support.

Coping, defined as efforts to manage stressors or associated emotional challenges (53), also plays a crucial role. Avoidant coping, characterized by disengagement or attempts to minimize consequences, is consistently associated with worse mental health, including anxiety (54, 55) and depression (56, 57). Among TGD individuals, coping moderates the effects of minority stress on mental health outcomes (25, 58, 59). Importantly, coping is multidimensional, involving both functional and dysfunctional strategies, and TGD individuals tend to adopt patterns distinct from cisgender populations due to the unique stressors they face (15, 60). Given these insights, our current study seeks to explore the roles of social support and coping in shaping mental health outcomes among treatment-seeking TGD individuals, thereby addressing critical gaps in the existing literature.

Since the establishment of the Gender Identity Clinic (GIC) in 2016, Hong Kong has provided centralized assessment and treatment for individuals experiencing gender incongruence. Within this framework, candidates for genital surgery must have undergone hormone therapy. They are also required to have lived in their desired gender role for a minimum of 12 months. These criteria ensure a stable transition process. Sin (61)’s study emerged as the largest local cohort to date, encompassing 394 TGD individuals registered with the GIC between 2019 and 2020 (61). Participants of different transition stages (i.e. having received varying degrees of gender-affirming treatments) were included through consecutive sampling. This approach allowed for comparisons of mood symptoms, gender congruence, social support, and coping.

Our follow-up study, conducted approximately 4 to 5 years after the initial cohort recruitment, captured the progression of participants in their transition journey. The research aimed to investigate whether gender-affirming treatments (i.e. GAS and GAHT) are associated with improvements in depressive and anxiety symptoms among TGD individuals while controlling for sociodemographic and clinical covariables. It also sought to explore whether gender-affirming treatments enhance gender congruence. Such insights are crucial for optimizing treatment approaches for service users at the GIC throughout their transition process.

## 2 Materials and methods

### 2.1 Design

This is a retrospective cohort follow-up study of 394 participants recruited during 2019 to 2020 by consecutive sampling at the GIC in Hong Kong. Throughout the follow-up data collection period (October 2023 to June 2024), those who attended the GIC were invited to participate in the present study. The inclusion criteria were participants who met the DSM-5 diagnosis of gender dysphoria, aged 18 or above, staying in Hong Kong who attended the GIC. Those without Chinese literacy skills or capability to give consent, have detransitioned (i.e. ceased to pursue transition and/or stopped identifying as transgender),have not received GAHT for at least 6 months at data collection or have discontinued GAHT for 6 months or more during the cohort were excluded. This study was approved by the Joint Chinese University of Hong Kong-New Territories East Cluster Clinical Research Ethics Committee (reference number: 2023.320). Written informed consent was obtained before their participation in the study. The participants were fully informed that they could withdraw from the study any time. Participants completed self-rated questionnaires to evaluate depressive and anxiety symptoms, along with gender incongruence.

### 2.2 Measures

#### 2.2.1 Socio-demographics and clinical information questionnaire

A self-report demographic questionnaire was distributed, including assigned sex at birth, identified gender, educational level, employment, living arrangement, and relationship status. Participants were asked to indicate the year of starting real-life experience (RLE);living full-time in their identified gender role, any active TGD community involvement, and all gender-affirming interventions received, including those performed outside of the public medical system. Case notes were reviewed to obtain relevant clinical information, including lifetime psychiatric comorbidities, active clinical psychologist care, and antidepressant use. The timings of initiating GAHT (including over-the-counter (OTC) prescriptions) and receiving GAS were also retrieved.

#### 2.2.2 The chinese version of depression, anxiety, and stress scale-21 items

The primary outcome of this study was measured by the DASS-21 (62). A higher score indicates higher level of emotional distress and poorer psychological adjustment. It consists of 3 subscales summing up each item rated from 0 (“Never”) to 3 (“Almost Always”). Severity is classified into normal, mild, moderate, and severe; cut-off for depression (10 or above), anxiety (8 or above), and stress (15 or above). The Chinese version of the DASS-21 (63) was translated and validated in Hong Kong, and was utilized in past local research studies. It demonstrated good internal consistency at baseline intake (α = .952).

#### 2.2.3 Gender congruence and llfe satisfaction scale (chinese version)

The GCLS (64) is a self-reported measure assessing physical, social, and psychological well-being of gender incongruence. 38 items are rated on a 5-point Likert-type scale. A higher score indicates better gender congruence, gender-related well-being, and greater life satisfaction. Questionnaire items are phrased in a way that applies to transmen, transwomen, and non-binary TGD individuals. The Chinese version was validated using data from patients in this initial cohort. It was demonstrated to have satisfactory validity, internal consistency (α = 0.929), and test-retest reliability. The Chinese GCLS yielded a 35-item, seven-factor model, which can be categorized into two subscales. The “Gender Congruence” subscale consists of genitalia, chest/breast, other secondary sexual characteristics, and social gender role recognition; whereas the “Gender Mental Wellbeing” cluster encompasses subscales that tap into mood symptoms, physical and emotional intimacy, and relationship satisfaction; wordings were intentionally crafted to assess gender-related issues instead of a generic measure of emotional distress. To be specific, the construct measures how often respondents experience feelings of low mood, anxiety, DSH, suicidal ideas, due to gender incongruence. It also reflects their overall satisfaction on functional aspects including social interactions, physical intimacy, work, and leisure activities.

#### 2.2.4 The chinese version of multidimensional scale of perceived social support

The MSPSS (65) is a self-administered measure of subjective adequacy of social support from family, friends, and significant others. 12-item ratings on a 7-point Likert- type scale, a higher score reflects more perceived support. This scale has been validated and is commonly used in the Hong Kong Chinese population (66). However, instead of three subscales, two major subscales emerged in the Chinese validation: the family subscale and the friends and significant other subscale. The Chinese version of the MSPSS was reported by Chou (66) to have good construct and concurrent validity, and good internal consistency as a whole (α = .89) and as individual subscales (family subscale α = .86; friends and significant others subscale α = .94).

#### 2.2.5 The Hong Kong chinese version of brief coping orientation to problems experienced inventory

The Brief COPE (67) is a 28-item measure derived from the original 60-item COPE Inventory (68). It evaluates strategies used to address, minimize, or tolerate stress associated with challenging or threatening events. The Hong Kong Chinese version of the Brief COPE was validated with an acceptable internal consistency for most items ranging from.64 to.87 (69). Respondents rated the frequency at a 4-point Likert scale (with “1” being “almost never” to “4” being “always”) of employing each type of coping responses. With reference to Tang et al. (69) specific subscales were selected for use in the analyses. In accordance with the 4-factor model proposed by Lindley and Bauerband (58), “active coping” and “interpersonal coping” represented facilitative coping strategies, whereas “cognitive avoidance” and “substance use” reflected avoidant coping strategies. Specifically, “facilitative coping” consisted of 9 items (including active coping, planning, positive reframing, using instrumental and emotional social support), while another 6 items (including substance use, behavioral disengagement, and self-blame) were categorized under “avoidant coping”. The internal consistencies for the “facilitative coping” and “avoidant coping” at baseline were α = .849 and α = .763 respectively.

### 2.3 Statistical analysis

Statistical analyses were performed using R (version 4.3.0). All tests were two-tailed (p < 0.05). A complete case analysis approach was adopted; missing data were excluded from the analysis.

Comparisons of sociodemographic questionnaire scores among the transition status groups (male-to-female, female-to-male, non-binary) were evaluated. Inter-group comparisons were conducted for time-invariant variables, and both inter-group and intra-group comparisons for time-variant variables. Statistically significant findings were followed up with *post-hoc* analyses using pairwise tests. A piecewise approach was adopted to account for the non-linear relationship of GAHT over time, with cut-off points defined at 2 years and 5 years (70).

Linear mixed-effects regression was adopted to test the longitudinal change in depressive and anxiety symptom scores among treatment groups. Outcome scores were log-transformed to meet the normality and equal variance assumptions if necessary.

Sensitivity analyses were conducted using linear regression, with the same covariates as the main regression model, while modifying the targeted variate. First, the duration of GAHT was recalculated by omitting over-the-counter hormone use (**Supplementary Table S1**). Second, analysis was performed after regrouping participants based on their transition direction (male-to-female, female-to-male, or non-binary (**Supplementary Table S2**). Third, regression models were rerun using only data from participants whose initial DASS-21 scores were above the normal cut-off, which consisted of around 40-50% of all participants (**Supplementary Table S3**).

## 3 Results

### 3.1 Description of participants

**Figure 1:**
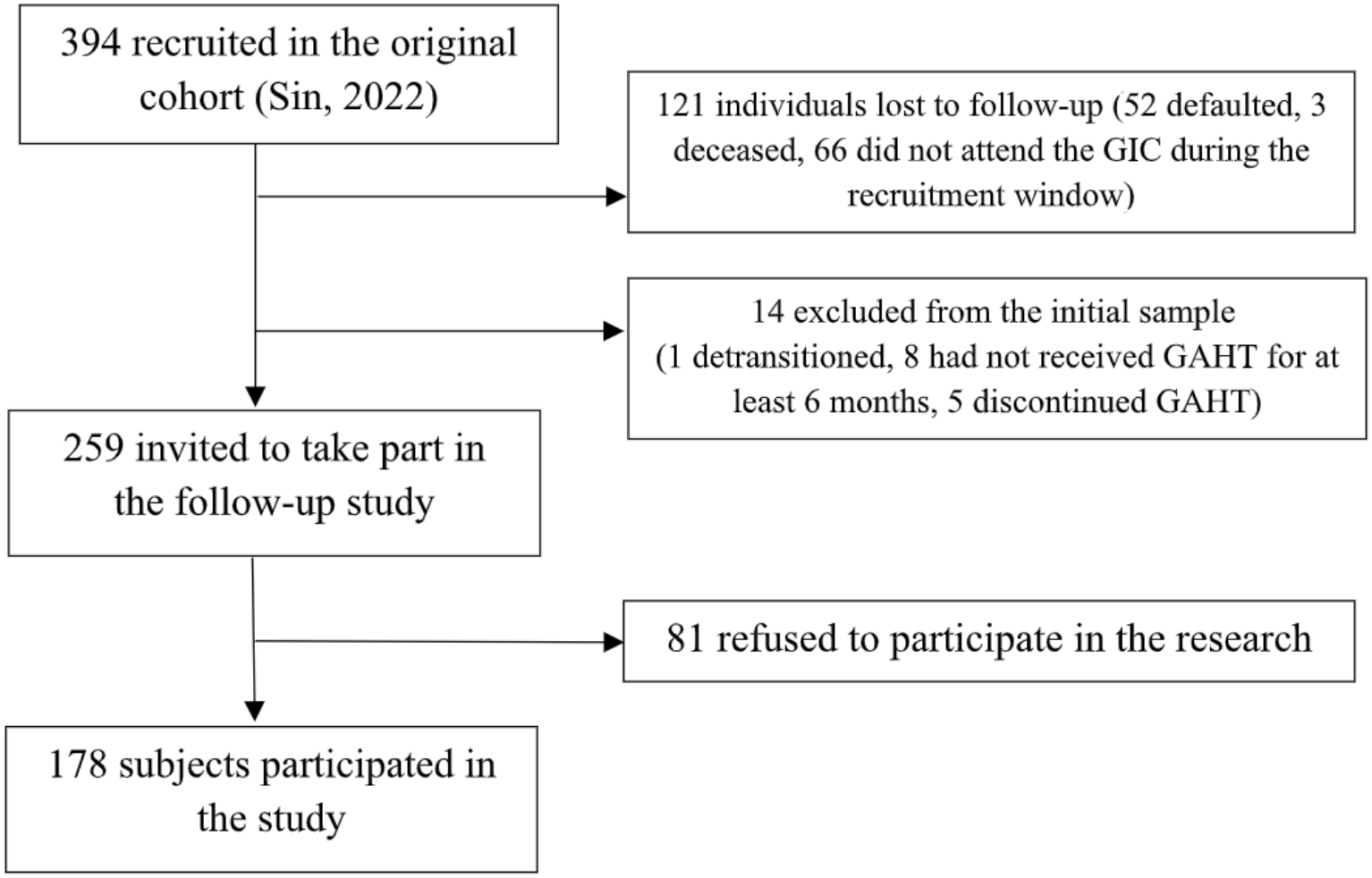
Study flow diagram.

The study sample was derived from an original cohort of 394 individuals (61). The final study sample comprised 178 participants, representing a response rate of 68.7% (**Figure 1**).

The sample consisted of 88 female-to-males (49.4%), 82 male-to-females (46.1%), and 8 non-binary participants (4.5%). No intersex subject was recruited. The median age was 36, 86.5% were single, 44.4% of them had tertiary education level or above, and 75.8% of them were employed or studying. More than half of them reported active involvement with the TGD community (52.8%). For clinical profile, 28.7% reported having lifetime psychiatric comorbidity, 25.3% were on antidepressants (or anxiolytics or antipsychotics), and 37.6% were receiving active care from clinical psychologists. At time of follow-up data collection (*t1*), the median durations of real-life experience (RLE) and GAHT were 9 and 7 respectively. 36.5% reported history of over-the-counter (OTC) hormone use.

### 3.2 Inter-group comparisons

Significant differences were identified among TGD status group (i.e., FtM versus MtF versus non-binary; p < 0.001), see **Tables 1a**, **b**. In particular, the “No GAS” (the group with no gender-affirming surgery) consisted of a majority of MtF individuals (82.5%) compared to other groups. The “GAS At Baseline” group reported the longest mean durations of GAHT (7.1 years; p < 0.001) and RLE (10.2 years; p < 0.001).

**Table 1a:** Descriptive analysis.

	No GAS (n=63)			GAS during cohort (n=53)			GAS at baseline (n=61)			Inter-group p-value	
	T0	T1	Intra-group p-value	T0	T1	Intra-group p-value	T0	T1	Intra-group p-value	T0	T1
Age (years)											
Mean (SD)	37.4 (10.51)			34.9 (8.68)			40.0 (7.89)			*0.005^k^	
Median [Min, Max]	36.0 [22, 73]			33.0 [22, 56]			38.0 [29, 69]				
Transition status											
Female-to-male	8			33			41				
Male-to-female	52			18			17			*<0.001^f^	
Non-binary	3			2			3				
Living status											
With others	42	50	0.653^c^	36	34	0.388^c^	44	43	0.334^c^	0.517^c^	0.215^c^
Alone	7	12		11	17		11	18			
Relationship status											
Single	43	56	0.901^c^	45	49	1.000^f^	47	48	0.482^c^	0.202^f^	*0.015^f^
Not single	6	6		2	2		8	13			
Education level											
Below tertiary	35	36	0.209^c^	25	27	1.000^c^	33	33	0.650^c^	0.177^c^	0.843^c^
Tertiary or above	14	26		22	24		22	28			
Employed, n (%)	19 (38.8)	22 (35.5)	0.874^c^	4 (8.5)	5 (9.8)	1.000^f^	9 (16.4)	12 (19.7)	0.825 ^c^	*<0.001^c^	*0.004^c^
Religious Belief, n (%)	16 (32.7)	20 (32.3)	1.000^c^	9 (19.1)	11 (21.6)	0.963^c^	19 (34.5)	20 (32.8)	0.997^c^	0.188^c^	0.352^c^
Active Trans Community, n (%)	31 (63.3)	27 (43.5)	0.061^c^	25 (53.2)	27 (52.9)	1.000^c^	34 (61.8)	37 (60.7)	1.000^c^	0.552^c^	0.164^c^
Aesthetic Procedures, n (%)	10 (20.4)	23 (37.1)	0.498^c^	8 (17.0)	14 (27.5)	0.241^c^	17 (33.3)	20 (32.8)	1.000^c^	0.563^c^	0.378^c^
Lifetime Psychiatric Comorbidity, n (%)	27 (42.9%)			17 (32.1%)			15 (24.6%)			0.095^c^	
Active Clinical Psychology, n (%)	43 (87.8)	33 (53.2)	*<0.001^c^	40 (85.1)	22 (43.1)	*<0.001^c^	18 (32.7)	11 (18.0)	0.107^c^	*<0.001^c^	*<0.001^c^
Antidepressant Use, n (%)	12 (24.5)	17 (27.4)	0.896^c^	7 (14.9)	13 (25.5)	0.294^c^	8 (14.5)	11 (18.0)	0.798^c^	0.340^c^	0.438^c^
RLE duration (years)											
Mean (SD)	4.4 (5.30)	8.4 (6.08)	*<0.001^w^	4.4 (6.99)	8.3 (7.09)	*<0.001^w^	10.2 (6.58)	14.2 (6.59)	*<0.001^w^	*<0.001^k^	*<0.001^k^
Median [Min, Max]	2.0 [0, 29]	7.0 [0, 33]		2.0 [0, 29]	6.0 [0, 33]		9.0 [0, 29]	13.0 [0, 33]			
GAHT duration (years)											
Mean (SD)	2.8 (4.48)	7.1 (4.47)	*<0.001^w^	2.0 (3.11)	5.9 (1.94)	*<0.001^w^	7,1 (4.85)	11.7 (4.67)	*<0.001^w^	*<0.001^k^	*<0.001^k^
Median [Min, Max]	1.0 [0, 22]	6.0 [1, 27]		1.0 [0, 16]	5.0 [3, 12]		7.0 [0, 22]	11.0 [4, 27]			

Active Trans Community, active transgender community involvement; GAS, gender-affirming surgery; GAHT, gender-affirming hormone therapy; RLE, real-life experience; T0, time-point 0; T1, time-point1; ^c^chi-square test; ^f^Fisher’s exact test; ^k^Kruskal-Wallis test; ^w^Wilcoxon rank-sum test; *p<0.05. Bold* refers to statistic signficance p<.005.

**Table 1b:** Descriptive analysis.

1		No GAS (n=63)			GAS during cohort (n=53)			GAS at baseline (n=61)			Inter-group p-value	
		T0	T1	Intra-group p-value	T0	T1	Intra-group p-value	T0	T1	Intra-group p-value	T0	T1
	Depression Score											
	Mean (SD)	14.2 (11.2)	15.5 (12.0)	0.584^w^	12.6 (11.6)	11.2 (9.7)	0.692^w^	8.9 (10.4)	10.4 (11.6)	0.604^w^	*0.018^k^	*0.016^k^
	Median [Min, Max]	12 [0, 40]	14 [0, 40]		8 [0, 42]	10 [0, 38]		6 [0, 40]	8 [0, 42]			
	Anxiety Score											
DASS-21	Mean (SD)	11.8 (8.4)	11.1 (8.6)	0.547^w^	10.5 (8.8)	9.0 (7.6)	0.471^w^	7.9 (8.4)	8.8 (8.8)	0.652^w^	*0.026^k^	0.179^k^
	Median [Min, Max]	12 [0, 28]	10 [0, 34]		10 [0, 36]	8 [0, 28]		4 [0, 36]	6 [0, 34]			
	Stress Score											
	Mean (SD)	16.2 (10.4)	16.6 (10.8)	0.861^w^	15.3 (10.9)	14.4 (10.9)	0.636^w^	12.4 (10.2)	13.6 (11.4)	0.752^w^	0.150^k^	0.247^k^
	Median [Min, Max]	16 [0, 38]	16 [0, 38]		14 [0, 38]	14 [0, 42]		12 [0, 38]	12 [0, 36]			
	Gender Congruence Score											
	Mean (SD)	49.9 (8.3)	48.9 (13.3)	0.610^x^	47.8 (7.3)	59.4 (11.4)	*<0.001^x^	56.1 (9.1)	63.9 (13.2)	*<0.001^x^	*<0.001^a^	*<0.001^a^
	Median [Min, Max]	50 [28, 73]	49 [17, 81]		46 [35, 64]	60 [29, 79]		56 [38, 82]	65 [36, 85]			
GCLS	Gender Mental Wellbeing Score											
	Mean (SD)	58.3 (10.2)	54.7 (13.1)	0.119^s^	58.6 (9.5)	60.6 (13.1)	0.377^w^	60.7 (8.3)	61.7 (13.9)	0.280^x^	0.337^a^	*0.007^k^
	Median [Min, Max]	59 [35, 75]	56 [23, 80]		60 [36, 78]	60 [26, 85]		62 [34, 75]	63 [22, 85]			
	Family Support Score											
	Mean (SD)	13.3 (6.4)	14.2 (6.9)	0.498^w^	13.9 (7.6)	16.1 (7.1)	0.119^w^	18.3 (7.2)	16.9 (8.4)	0.375^w^	*<0.001^k^	0.148^k^
	Median [Min, Max]	13 [4, 28]	15 [4, 27]		14 [4, 28]	16 [4, 28]		19 [4, 28]	18 [4, 28]			
MSPSS	Friends and SO Support Score											
	Mean (SD)	36.1 (14.3)	34.4 (14.3)	0.513^w^	39.0 (13.7)	36.3 (15.5)	0.411^w^	42.6 (15.0)	40.9 (15.2)	0.644^w^	*0.020^k^	*0.019^k^
	Median [Min, Max]	36 [8, 56]	38 [8, 56]		43 [8, 56]	40 [8, 56]		48 [8, 56]	45 [8, 56]			
	Facilitative Coping Score											
	Mean (SD)	24.2 (4.1)	23.5 (3.7)	0.355^s^	25.3 (5.5)	24.8 (4.5)	0.597^s^	27.0 (5.3)	26.5 (4.7)	0.559^s^	*0.010 ^k^	*<0.001^k^
Brief	Median [Min, Max]	24 [11, 34]	23 [17, 34]		25 [11, 36]	24 [14, 35]		27 [12, 36]	27 [13,36]			
COPE	Avoidant Coping Score											
	Mean (SD)	12.5 (3.2)	12.5 (3.4)	0.942^w^	12.4 (3.7)	11.7 (3.2)	0.459^w^	12.4 (4.0)	12.5 (3.6)	0.547^w^	0.666^k^	0.329^k^
	Median [Min, Max]	13 [6, 19]	13 [6, 22]		12 [6, 23]	11 [6, 22]		12 [6, 24]	13 [6, 24]			

Brief COPE, Brief Coping Orientation to Problems Experienced Inventory; DASS-21, Depression, Anxiety, and Stress Scale-21 items; GCLS, Gender Congruence and Life Satisfaction Scale; MSPSS, Multidimensional Scale of Perceived Social Support; Friends and SO Support Score, Friends and Significant Others Support Score; T0, time-point 0; T1, time-point1; ^k^Kruskal-Wallis test; ^s^Student’s t-test; ^w^Wilcoxon rank-sum test; ^x^Welch’s t-test; *p<0.05. Bold* refers to statistic signficance p<.005.

No significant differences were observed regarding the presence of lifetime psychiatric diagnoses, antidepressant use, aesthetic procedures, living status, relationship status, and education level. The comparing groups did not differ significantly in terms of sociodemographic profile. The “GAS At Baseline” group was characterized by the mildest levels of depressive (p = 0.018) and anxiety symptoms (p = 0.026), and highest level of gender congruence (p < 0.001). The same group also scored highest in family support (p < 0.001), friends and significant other support (p = 0.020), and facilitative coping (p = 0.010). Causal inference cannot be drawn from a cross-sectional analysis of data. However, it is noteworthy that the post-GAS gender congruence scores of the “GAS During Cohort” group increased to a level similar to the baseline results of those who had received GAS at intake.

### 3.3 Intra-group comparisons (baseline versus follow-up)

No significant differences were observed within any of the groups concerning depressive, anxiety, and stress, coping and social support scores over time, see **Tables 2a**, **b**.

**Table 2a:** Regression analysis of depression model (GAS grouping).

Variable	Estimate (95% CI)	p-value
Time [1]	1.144 (-2.713 – 5.001)	0.563
Group [GAS During Cohort]	0.207 (-3.630 – 4.044)	0.916
GAHT Duration by Year 2	-0.164 (-2.265 – 1.937)	0.879
GAHT Duration Year 2-5	-0.076 (-1.584 – 1.433)	0.922
GAHT Duration after Year 5	-0.220 (-0.687 – 0.246)	0.358
Family Support	-0.240 (-0.433 – -0.047)	*0.017
Friends and Significant Others Support	-0.135 (-0.231 – -0.039)	*0.007
Facilitative Coping	-0.426 (-0.723 – -0.128)	*0.006
Avoidant Coping	1.377 ( 0.989 – 1.765)	*<0.001
Time [1] x Group [GAS During Cohort]	-0.765 (-4.826 – 3.296)	0.713

Random Intercept Variance = 20.9; Residual Variance = 44.8

GAS, gender-affirming surgery; GAHT, gender-affirming hormone therapy; *p<0.05. Bold* refers to statistic signficance p<.005.

**Table 2b:** Regression analysis of depression model (GAHT grouping).

Variable	Estimate (95% CI)	p-value	AME
Time [1]	1.041 (0.847 – 1.280)	0.703	0.25
Group [GAHT During Cohort]	1.197 (0.836 – 1.713)	0.329	1.22
Chest Surgery [Yes]	0.921 (0.628 – 1.351)	0.675	-0.49
Genital Surgery [Yes]	1.075 (0.815 – 1.418)	0.611	0.46
Family Support	1.021 (1.006 – 1.037)	*0.009	0.24
Friends and Significant Others Support	1.013 (1.004 – 1.022)	*0.003	0.4
Facilitative Coping	0.957 (0.933 – 0.982)	*0.001	-0.77
Avoidant Coping	1.104 (1.068 – 1.141)	*<0.001	0.5
Time [1] x Group [GAHT During Cohort]	0.927 (0.653 – 1.318)	0.675	-0.51

Random Intercept Variance = 0.33; Residual Variance = 0.46

GAHT, gender-affirming hormone therapy; *p<0.05. Bold* refers to statistic signficance p<.005.

During the follow-up period, depressive symptoms declined in the “GAS During Cohort” group, whereas they increased in the other two groups. Similarly, anxiety symptoms decreased over time in the “GAS During Cohort” group but showed an increase in the “GAS At Baseline” group.

Of note is the significant longitudinal improvement in mean gender congruence scores for the 2 groups that received GAS (i.e. “GAS During Cohort”, p < 0.001; “GAS At Baseline”, p < 0.001).

### 3.4 Regression analysis

For the major analyses based on GAS status grouping, the “GAS At Baseline” group was excluded because it provided no additional information in the pre-and-post GAS comparison. Addition analyses were conducted according to a separate grouping by GAHT status, with additional GAS controlled as covariables. Average marginal effect (AME) was reported for log-transformed models, representing the average change in the outcome score for a one-unit change in the independent variable. Other model coefficients are reported in absolute score change.

#### 3.4.1 Depression

Regression analysis showed that neither GAS nor GAHT posed a significant effect on depressive symptoms (**Table 3a**). Family support (β = -0.240, p = 0.017) and friends and significant other support (β = -0.135, p = 0.007) were associated with less depressive symptoms. Facilitative coping was associated with a decrease in depressive symptoms (β = -0.426, p = 0.006), while avoidant coping was associated with worsened depressive symptoms (β = 1.377, p < 0.001). Similar associations were found using the GAHT grouping regression model (**Table 3b**).

**Table 3a:** Regression analysis of log-anxiety model (GAS grouping).

Variable	Estimate (95% CI)	p-value	AME
Time [1]	1.046 (0.702 – 1.559)	0.826	0.32
Group [GAS During Cohort]	0.915 (0.604 – 1.388)	0.678	-0.58
GAHT Duration by Year 2	0.994 (0.803 – 1.232)	0.960	-0.04
GAHT Duration Year 2-5	0.949 (0.814 – 1.108)	0.511	-0.37
GAHT Duration after Year 5	0.984 (0.935 – 1.036)	0.539	-0.12
Family Support	0.984 (0.964 – 1.004)	0.124	-0.23
Friends and Significant Others Support	0.995 (0.985 – 1.005)	0.332	-0.18
Facilitative Coping	0.971 (0.940 – 1.002)	0.073	-0.50
Avoidant Coping	1.089 (1.045 – 1.134)	*<0.001	0.49
Time [1] x Group [GAS During Cohort]	1.163 (0.774 – 1.748)	0.470	1.05

Random Intercept Variance = 0.34; Residual Variance = 0.43

GAS, gender-affirming surgery; GAHT, gender-affirming hormone therapy; *p<0.05. Bold* refers to statistic signficance p<.005.

**Table 3b:** Regression analysis of log-anxiety model (GAHT grouping).

Variable	Estimate (95% CI)	p-value	AME
Time [1]	1.019 (0.842 – 1.232)	0.849	0.08
Group [GAHT During Cohort]	1.201 (0.847 – 1.704)	0.305	0.87
Chest Surgery [Yes]	1.348 (0.921 – 1.973)	0.126	1.51
Genital Surgery [Yes]	0.953 (0.723 – 1.257)	0.734	-0.2
Family Support	1.012 (0.997 – 1.027)	0.127	0.1
Friends and Significant Others Support	1.009 (1.001 – 1.017)	*0.035	0.19
Facilitative Coping	0.962 (0.939 – 0.986)	*0.002	-0.45
Avoidant Coping	1.091 (1.057 – 1.126)	*<0.001	0.31
Time [1] x Group [GAHT During Cohort]	0.916 (0.667 – 1.259)	0.591	-0.43

Random Intercept Variance = 0.38; Residual Variance = 0.37

GAHT, gender-affirming hormone therapy; *p<0.05. Bold* refers to statistic signficance p<.005.

#### 3.4.2 Anxiety

GAS or GAHT showed no significant effects on anxiety symptoms (**Tables 1a**, **b**). Avoidant coping was associated with higher anxiety scores (p < 0.001).

#### 3.4.3 Stress

GAS or GAHT showed no significant effects on stress scores (**Tables 4a**, **b**). Avoidant coping increased stress symptoms (p < 0.001). Friends and significant other support were associated with lower stress scores (p = 0.015).

**Table 4a:** Regression analysis of stress model (GAS grouping).

Variable	Estimate (95% CI)	p-value
Time [1]	2.389 ( -1.686 – 6.464)	0.254
Group [GAS During Cohort]	1.193 ( -2.777 – 5.163)	0.557
GAHT Duration by Year 2	0.209 ( -2.021 – 2.439)	0.855
GAHT Duration Year 2-5	-0.988 ( -2.587 – 0.611)	0.230
GAHT Duration after Year 5	-0.019 ( -0.499 – 0.462)	0.940
Family Support	-0.086 ( -0.287 – 0.116)	0.407
Friends and Significant Others Support	-0.128 (-0.229 – -0.028)	*0.015
Facilitative Coping	-0.103 ( -0.412 – 0.206)	0.516
Avoidant Coping	1.270 ( 0.865 – 1.675)	*<0.001
Time [1] x Group [GAS During Cohort]	0.513 ( -3.834 – 4.860)	0.818

Random Intercept Variance = 18.1; Residual Variance = 52.2

GAS, gender-affirming surgery; GAHT, gender-affirming hormone therapy; *p<0.05. Bold* refers to statistic signficance p<.005.

**Table 4b:** Regression analysis of stress model (GAHT grouping).

Variable	Estimate (95% CI)	p-value	AME
Time [1]	1.032 (0.838 – 1.271)	0.765	0.25
Group [GAHT During Cohort]	1.097 (0.785 – 1.533)	0.588	0.75
Chest Surgery [Yes]	1.057 (0.750 – 1.489)	0.753	0.44
Genital Surgery [Yes]	1.138 (0.888 – 1.457)	0.308	1.06
Family Support	1.012 (0.997 – 1.027)	0.111	0.18
Friends and Significant Others Support	1.008 (1.000 – 1.016)	*0.046	0.32
Facilitative Coping	0.948 (0.960 – 1.008)	0.186	-0.29
Avoidant Coping	1.105 (1.071 – 1.140)	*<0.001	0.62
Time [1] x Group [GAHT During Cohort]	1.075 (0.748 – 1.547)	0.696	0.68

Random Intercept Variance = 0.19; Residual Variance = 0.50

GAHT, gender-affirming hormone therapy; *p<0.05. Bold* refers to statistic signficance p<.005.

#### 3.4.4 Gender congruence

No significant interaction with time was found in the “No GAS” group (p = 0.289), indicating that gender congruence remained stable over time. However, individuals in the “GAS During Cohort” group showed an average increase of 11.99 points in gender congruence (p < 0.001) compared to the “No GAS” group. With the full score being 65 (13 items measuring gender congruence on the GCLS), an increase of 11.99 points can be considered as a large and clinically meaningful effect (**Table 5a**). In other words, the results indicated a large and clinically meaningful improvement in gender congruence after gender-affirming treatments.

**Table 5a:** Regression analysis of gender congruence model (GAS grouping).

Variable	Estimate (95% CI)	P-value
Time [1]	-1.414 (-6.420 – 3.592)	0.581
Group [GAS During Cohort]	-2.737 (-7.502 – 2.028)	0.263
GAHT Duration by Year 2	-1.141 (-3.891 – 1.609)	0.419
GAHT Duration Year 2-5	1.354 (-0.614 – 3.322)	0.181
GAHT Duration after Year 5	-0.077 (-0.648 – 0.495)	0.794
Family Support	0.004 (-0.240 – 0.247)	0.976
Friends and Significant Others Support	0.129 ( 0.007 – 0.251)	*0.042
Facilitative Coping	-0.230 (-0.600 – 0.141)	0.228
Avoidant Coping	-0.220 (-0.709 – 0.269)	0.381
Time [1] x Group [GAS During Cohort]	11.992 ( 6.570 – 17.414)	*<0.001

Random Intercept Variance = 18.6; Residual Variance = 82.9

GAS, gender-affirming surgery; GAHT, gender-affirming hormone therapy; *p<0.05. Bold* refers to statistic signficance p<.005.

**Table 5b:** Regression analysis of gender congruence model (GAHT grouping).

Variable	Estimate (95% CI)	P-value
Time [1]	6.592 (3.740 – 9.445)	*<0.001
Group [GAHT During Cohort]	0.634 (-3.752 – 5.020)	0.777
Chest Surgery [Yes]	0.597 (-3.781 – 4.975)	0.79
Genital Surgery [Yes]	4.346 (1.194 – 7.498)	*0.008
Family Support	-0.086 (-0.281 – 0.109)	0.390
Friends and Significant Others Support	-0.114 (-0.220 – -0.008)	*0.037
Facilitative Coping	0.072 (-0.247 – 0.390)	0.66
Avoidant Coping	-0.380 (-0.789 – 0.029)	0.071
Time [1] x Group [GAHT During Cohort]	5.175 (0.126 – 10.225)	*0.047

Random Intercept Variance = 20.64; Residual Variance = 97.12

GAHT, gender-affirming hormone; *p<0.05. Bold* refers to statistic signficance p<.005.

**Table 5c:** Regression analysis of chest/breast congruence model (GAS grouping).

Variable	Estimate (95% CI)	P-value
Time [1]	0.084 (-1.020 – 1.188)	0.882
Group [GAS At Baseline]	2.306 (0.914 – 3.699)	*0.001
Group [GAS During Cohort]	-0.919 (-2.120 – 0.281)	0.135
Transition status (American Psychiatric Association & Association)	1.243 ( 0.317 – 2.169)	*0.009
GAHT Duration by Year 2	-0.259 (-0.891 – 0.372)	0.422
GAHT Duration Year 2-5	0.601 (0.205 – 0.996)	*0.003
GAHT Duration after Year 5	-0.079 (-0.189 – 0.032)	0.165
Time [1] x Group [GAS At Baseline]	0.547 (-0.716 – 1.810)	0.398
Time [1] x Group [GAS During Cohort]	2.527 (1.267 – 3.787)	*<0.001

Random Intercept Variance = 3.00; Residual Variance = 4.36

GAS, gender-affirming surgery; GAHT, gender-affirming hormone therapy; MtF, male-to-female; *p<0.05. Bold* refers to statistic signficance p<.005.

The regression analysis which adopted the GAHT status grouping also revealed significant findings (**Table 5b**) compared to the trend in the “GAHT At Baseline” (p = 0.047). Longitudinal improvement in gender congruence was noted in both groups. The “GAHT At Baseline” group scored an average increase of 6.59 points over time (p < 0.001), but the magnitude of improvement was even larger in the “GAHT During Cohort” group (p = 0.047). Furthermore, receiving genital surgery improved gender congruence scores in this model (p = 0.008).

Further analyses were conducted using the four subscales constituent of the overall gender congruence scores (i.e. genitalia, chest/breast, other secondary sexual characteristics, and social gender role recognition) as independent outcomes. GAHT duration of 2-to-5 years was found to improve chest/breast congruence (p = 0.003; **Table 5c**). Also, identifying as male-to-female was associated with significantly better chest/breast congruence (p = 0.009). No statistically significant correlation was observed in other subscales with reference to GAHT use.

#### 3.4.5 Gender mental wellbeing

No significant association was found for GAS or GAHT with gender mental wellbeing, see **Tables 6a**, **b**. Factors that improved gender mental wellbeing included family support (p = 0.009) and friends and significant other support (p = 0.006). Avoidant coping was found to worsen gender mental wellbeing (p < 0.001).

**Table 6a:** Regression analysis of gender mental wellbeing model (GAS grouping).

Variable	Estimate (95% CI)	P-value
Time [1]	-2.683 ( -7.052 – 1.687)	0.233
Group [GAS During Cohort]	-3.789 ( -7.788 – 0.210)	0.066
GAHT Duration by Year 2	1.949 ( -0.457 – 4.355)	0.117
GAHT Duration Year 2-5	-0.949 ( -2.664 – 0.767)	0.282
GAHT Duration after Year 5	-0.012 ( -0.483 – 0.460)	0.962
Family Support	0.282 ( 0.077 – 0.486)	*0.009
Friends and Significant Others Support	0.148 ( 0.046 – 0.251)	*0.006
Facilitative Coping	0.292 ( -0.016 – 0.600)	0.067
Avoidant Coping	-1.095 ( -1.507 – -0.683)	*<0.001
Time [1] x Group [GAS During Cohort]	4.055 ( -0.806 – 8.916)	0.106

Random Intercept Variance = 3.2; Residual Variance = 69.1

GAS, gender-affirming surgery; GAHT, gender-affirming hormone therapy; *p<0.05. Bold* refers to statistic signficance p<.005.

**Table 6b:** Regression analysis of gender mental wellbeing model (GAHT grouping).

Variable	Estimate (95% CI)	P-value
Time [1]	0.835 (-1.449 – 3.119)	0.475
Group [GAHT During Cohort]	-1.885 (-5.292 – 1.523)	0.280
Chest Surgery [Yes]	-2.066 (-5.396 – 1.264)	0.226
Genital Surgery [Yes]	-0.059 (-2.453 – 2.334)	0.961
Family Support	-0.242 (-0.394 – -0.091)	*0.002
Friends and Significant Others Support	-0.132 (-0.215 – -0.050)	*0.002
Facilitative Coping	0.343 (0.097 – 0.590)	*0.007
Avoidant Coping	-1.099 (-1.416 – -0.783)	*<0.001
Time [1] x Group [GAHT During Cohort]	-0.667 (-4.746 – 3.413)	0.749

Random Intercept Variance = 7.51; Residual Variance = 63.94

GAHT, gender-affirming hormone therapy; *p<0.05. Bold* refers to statistic signficance p<.005.

### 3.5 Sensitivity analyses

Firstly, the duration of GAHT was recalculated by omitting over-the-counter hormone use. The analysis showed a similar pattern of correlations, in which gender-affirming treatments were not associated with changes in mental health scores. These results demonstrate the robustness of the main regression findings (Appendix: **Supplementary Table S1**). Secondly, analysis was performed after regrouping participants based on their transition direction (male-to-female, female-to-male, or non-binary). Results revealed that identifying as non-binary was significantly associated with worsened depressive (p = 0.004) and stress symptoms (p = 0.037) over time compared to the FtM reference group. Conversely, identifying as MtF had no significant associations with any psychological outcomes (**Supplementary Table S2**). Thirdly, regression models were rerun using only data from participants whose initial DASS-21 scores were above the normal cut-off, which consisted of around 40-50% of all participants. In this subgroup with worse symptoms at baseline, symptom improvement was shown to be significantly associated with coping and family support, but not GAS or GAHT (**Supplementary Table S3**).

## 4 Discussion

The present study is the largest cohort to date to investigate the longitudinal outcome of gender-affirming treatments in the TGD population in Hong Kong. This study primarily aimed to examine whether gender-affirming surgeries (GAS) and gender-affirming hormone therapy (GAHT) reduces depressive and anxiety symptoms, and secondarily, whether such treatments reduce gender incongruence. We found that while GAS and GAHT both improve gender congruence, they did not significantly reduce depressive or anxiety symptoms. Instead, significant associations with mood symptoms were found for social support and coping.

### 4.1 Depressive, anxiety, and stress symptoms

Gender-affirming treatments were found not to worsen depressive or anxiety symptoms, consistent with existing literature, but no significant improvements were observed either. This may be attributed to the dynamic nature of gender transition, where the initiation of GAHT presents unique challenges, such as the variable onset of physical changes. The psychological benefits of GAS and GAHT depend largely on how closely a TGD individual’s appearance aligns with societal norms for cisgender individuals. For instance, while testosterone therapy masculinizes certain features, breast size may remain unchanged. These persistent social challenges could contribute to continued depressive or anxious symptoms. In addition, our findings indicate that social gender-role recognition is not directly associated with GAS/GAHT. This suggests that if medical interventions do not lead to greater recognition in daily social contexts, individual may continue to face to stigma and discrimination, which could help explain the persistence of mood symptoms.

Additionally, long-term psychological well-being may be impacted by GAHT-related medical comorbidities (e.g., increased cardiovascular risk (71), aesthetic outcomes of GAS, and potential postoperative complications. Moreover, gender-affirming treatments alone may not address the mental health challenges that persist post-intervention (38, 72), as individuals may continue to face societal micro- and macro-aggressions, internalized stigma, and experiences of misgendering (4, 27, 73).

The treatment-seeking sample’s composition may have influenced results, as participants were drawn from a specialized clinic where psychiatric evaluation and psychological counseling were provided. Approximately a quarter had previously used antidepressants or anxiolytics, which could obscure the effects of gender-affirming interventions (38). Additionally, without a cisgender or non-treatment-seeking TGD control group, any rise in co-occurring mental health issues during the study, potentially exacerbated by stressors such as the COVID-19 pandemic (64, 74), could not be accounted for. The linear mixed-effects regression model used in this study is a strength, as it simultaneously considers fixed effects (treatment received) and random effects (individual differences), allowing for a more accurate assessment of treatment effects compared to logistic or multiple linear regression methods.

### 4.2 Gender congruence and gender mental wellbeing

GAS and GAHT were significantly associated with increased gender congruence, supporting the hypothesis that gender-affirming treatments enhance body satisfaction and alleviate gender incongruence. Our results showed that chest/breast congruence improved to a greater extent than other areas. This aligns with current understanding that maximum benefits from GAHT on physical outlook, namely body fat redistribution and breast growth, are typically achieved over a 2 to 5-year window (70). However, it should be noted that other aspects of GAHT-induced physical changes, such as voice deepening and facial or body hair growth, may not be fully captured by the GCLS. Specifically, the scale only consisted of 2 items assessing gender congruence relating to hair distribution, and another 1 item relating to voice, together grouped as “other secondary sexual characteristics”. This may explain why no significant association was yielded between GAHT and longitudinal improvement in the “other secondary sexual characteristics” subscale. In addition, no significant improvements were observed beyond 5 years, suggesting a potential “ceiling effect” (38). Some GAHT-induced changes, such as voice deepening and hair growth, may not be fully captured by the GCLS, which includes limited items on secondary sexual characteristics.

In this study, Wellbeing scores showed moderate to high negative correlation with DASS-21 scores (0.6-0.7); and a moderate correlation with MSPSS scores (0.4-0.5). Our study results are in line with previous studies which observed that mental wellbeing did not improve despite greater gender congruence (38, 75). Several factors may contribute to this observation. Psychosocially, they may continue to face micro- and macro-aggressions in society. Deeply ingrained negative self-perceptions and internalized stigma may persist, manifesting as high levels of distress (76). In some cases, experiences of misgendering and rejection may even increase briefly after initiating GAHT (4) due to heightened expectations and desire to “pass” within a cisnormative binary gender framework (i.e., visual conformity with affirmed gender (27). Further research is needed to shed light on any temporal relationship in transition outcomes, for instance, whether mental wellbeing lag behind body congruence improvements. Our regression analyses support the notion that while gender congruence is significantly associated with gender-affirming treatments, improvements in gender congruence do not necessarily reflect in general psychological measures, highlighting the need for tailored assessments in TGD healthcare (72).

### 4.3 Roles of coping and social support

Regression analyses indicated that coping mechanisms, particularly avoidant and facilitative strategies, played a stronger role than gender-affirming treatments in predicting mood improvements among TGD individuals. According to the gender minority stress model (15), avoidant coping has been shown to mediate the psychological impact of minority stress, as ongoing threats of discrimination can sustain distress and undermine self-efficacy (25, 59), while cognitive and behavioral avoidance limit positive reinforcement and contribute to depression (54, 77). Conversely, facilitative coping, including active information seeking and cognitive reframing can enhance self-efficacy and self-acceptance while buffering against the negative impact of discrimination among gender minorities (78, 79).

Social support also emerged as a protective factor, enhancing quality of life (8), reducing depressive symptoms (41, 42)and suicidal ideation (80), and improving well-being (81). However, the commonly used MSPSS scale has been criticized for cisnormativity, failing to account for the importance of TGD peer connections (82). In line with this, the current study found that active TGD community involvement was associated with lower depressive symptoms and stress, albeit not at significant levels, highlighting the need for a more tailored social support measure for TGD adults.

### 4.4 Sociodemographic and clinical characteristics

Our results suggest that FtM individuals often experience smoother social transitions and greater congruence with their physical appearance compared to MtF individuals, partly due to societal norms that place a higher threshold on “passing” as male (31, 37). Transwomen, therefore, may face greater marginalization, reporting higher rates of discrimination and lower family support (46, 83). However, a recent systematic review found no consistent outcome differences between MtF and FtM groups, a finding supported by our current study (38).

### 4.5 Limitations and future research

Several limitations of the research should be considered when interpreting the results. This study is limited by sampling bias from treatment-seeking individuals, potential confounding effects of integrated psychosocial care, and analytic constraints such as grouping different hormone regimens and surgeries, lack of data on surgical desire, and absence of standardized measures for community support. Additional limitations include unmeasured clinical variables (e.g., antidepressant dosage, psychological follow-up) and differential attrition, which may reduce representativeness.

Future research should adopt larger, prospective designs with longer follow-ups, use validated tools to assess gender congruence, and incorporate psychosocial factors, minority stress, and community support, while ensuring inclusivity of non-binary and diverse TGD populations.

## 5 Conclusions

This study addresses a critical gap in transgender healthcare by examining the relationship between gender-affirming treatments and mental health outcomes. The findings reveal that while gender-affirming surgeries and hormone therapy significantly enhance gender congruence, they do not directly translate to reductions in depressive or anxiety symptoms. This highlights the persistence of underlying psychosocial challenges during the transition journey, which cannot be universally resolved through medical interventions alone. Healthcare providers should set realistic expectations and offer comprehensive guidance, while mental health professionals play a crucial role in supporting the coming-out process, social transitioning, and addressing maladaptive cognitions, behaviors, and interpersonal deficits.

By emphasizing the need for holistic care, this study advances knowledge in transgender health and reinforces the importance of integrating medical and psychosocial support. These findings align with current guidelines but also highlight the necessity of addressing the broader psychosocial dimensions of well-being. Clinically, they advocate for multidisciplinary care models that combine medical treatments with mental health support, while future research should explore targeted interventions to better meet the diverse needs of this marginalized population.

## Data Availability

The raw data supporting the conclusions of this article will be made available by the authors, without undue reservation.

## References

[1] Wittlin NM Kuper LE Olson KR . Mental health of transgender and gender diverse youth. Annu Rev Clin Psychol. (2023) 19:207–32. doi: 10.1146/annurev-clinpsy-072220-020326, PMID: 36608332 PMC9936952

[2] Association., A. P . Diagnostic and statistical manual of mental disorders. 4th ed. K Street, N.W., Washington, DC: American Psychiatric Publishing, Inc (1994).

[3] Association, A. P . Diagnostic and statistical manual of mental disorders: DSM-5™. 5th ed. K Street, N.W., Washington, DC: American Psychiatric Publishing, Inc (2013). doi: 10.1176/appi.books.9780890425596

[4] Robles R Fresán A Vega-Ramírez H Cruz-Islas J Rodríguez-Pérez V Domínguez-Martínez T . Removing transgender identity from the classification of mental disorders: a Mexican field study for ICD-11. Lancet Psychiatry. (2016) 3:850–9. doi: 10.1016/S2215-0366(16)30165-1, PMID: 27474250

[5] Moagi MM van der Wath AE Jiyane PM Rikhotso RS . Mental health challenges of lesbian, gay, bisexual and transgender people: An integrated literature review. Health SA Gesondheid. (2021) 26. doi: 10.4102/hsag.v26i0.1487, PMID: 33604059 PMC7876969

[6] Witcomb GL Bouman WP Claes L Brewin N Crawford JR Arcelus J . Levels of depression in transgender people and its predictors: Results of a large matched control study with transgender people accessing clinical services. J Affect Disord. (2018) 235:308–15. doi: 10.1016/j.jad.2018.02.051, PMID: 29665513

[7] Bouman WP Claes L Marshall E Pinner GT Longworth J Maddox V . Sociodemographic variables, clinical features, and the role of preassessment cross-sex hormones in older trans people. J Sexual Med. (2016) 13:711–9. doi: 10.1016/j.jsxm.2016.01.009, PMID: 26897462

[8] Davey A Bouman WP Arcelus J Meyer C . Social support and psychological well-being in gender dysphoria: A comparison of patients with matched controls. J Sexual Med. (2014) 11:2976–85. doi: 10.1111/jsm.12681, PMID: 25155247

[9] Paz-Otero M Becerra-Fernández A Pérez-López G Ly-Pen D . A 2020 review of mental health comorbidity in gender dysphoric and gender non-conforming people. J Psychiatry Treat Res. (2021) 3:44–55.

[10] Millet N Longworth J Arcelus J . Prevalence of anxiety symptoms and disorders in the transgender population: A systematic review of the literature. Int J Transgenderism. (2017) 18:27–38. doi: 10.1080/15532739.2016.1258353

[11] Pellicane MJ Ciesla JA . Associations between minority stress, depression, and suicidal ideation and attempts in transgender and gender diverse (TGD) individuals: Systematic review and meta-analysis. Clin Psychol Rev. (2022) 91:102113. doi: 10.1016/j.cpr.2021.102113, PMID: 34973649

[12] Suen YT Chan RCH Wong EMY . Excluded lives: The largest scale survey on the social and legal marginalisation of transgender people in Hong Kong so far (2021). Hong Kong. Available online at: https://tgr.org.hk/attachments/article/340/TransReport%2020210512_English.pdf (Accessed May 15, 2025).

[13] Carmel TC Erickson-Schroth L . Mental health and the transgender population. J Psychosocial Nurs Ment Health Serv. (2016) 54:44–8. doi: 10.3928/02793695-20161208-09, PMID: 28001287

[14] Riggs DW Ansara GY Treharne GJ . An evidence-based model for understanding the mental health experiences of transgender Australians. Aust Psychol. (2015) 50:32–9. doi: 10.1111/ap.12088

[15] Hendricks ML Testa RJ . A conceptual framework for clinical work with transgender and gender nonconforming clients: An adaptation of the Minority Stress Model. Prof Psychology: Res Pract. (2012) 43:460–7. doi: 10.1037/a0029597

[16] Meyer IH . Prejudice, social stress, and mental health in lesbian, gay, and bisexual populations: Conceptual issues and research evidence. psychol Bull. (2003) 129:674–97. doi: 10.1037/0033-2909.129.5.674, PMID: 12956539 PMC2072932

[17] Coleman E Radix AE Bouman WP Brown GR De Vries ALC Deutsch MB . Standards of care for the health of transgender and gender diverse people, version 8. Int J Transgender Health. (2022) 23:S1–S259. doi: 10.1080/26895269.2022.2100644, PMID: 36238954 PMC9553112

[18] Baker KE Wilson LM Sharma R Dukhanin V McArthur K Robinson KA . Hormone therapy, mental health, and quality of life among transgender people: A systematic review. J Endocrine Soc. (2021) 5:bvab011. doi: 10.1210/jendso/bvab011, PMID: 33644622 PMC7894249

[19] Rowniak S Bolt L Sharifi C . Effect of cross-sex hormones on the quality of life, depression and anxiety of transgender individuals: a quantitative systematic review. JBI Database Systematic Rev Implementation Rep. (2019) 17:1826–54. doi: 10.11124/JBISRIR-2017-003869, PMID: 31021971

[20] Doyle DM Lewis TOG Barreto M . A systematic review of psychosocial functioning changes after gender-affirming hormone therapy among transgender people. Nat Hum Behav. (2023) 7:1320–31. doi: 10.1038/s41562-023-01605-w, PMID: 37217739 PMC10444622

[21] Nobili A Glazebrook C Arcelus J . Quality of life of treatment-seeking transgender adults: A systematic review and meta-analysis. Rev Endocrine Metab Disord. (2018) 19:199–220. doi: 10.1007/s11154-018-9459-y, PMID: 30121881 PMC6223813

[22] Das HK Kanmani TR Nagamangala PN Kalra P Rajendaran S . Impact of gender-affirming interventions on mental health and body image satisfaction of transgender individuals: A systematic review. Indian J Endocrinol Metab. (2025) 29:484–94. doi: 10.4103/ijem.ijem_24_25, PMID: 41229723 PMC12604846

[23] Van Leerdam TR Zajac JD Cheung AS . The effect of gender-affirming hormones on gender dysphoria, quality of life, and psychological functioning in transgender individuals: A systematic review. Transgender Health. (2023) 8:6–21. doi: 10.1089/trgh.2020.0094, PMID: 36895312 PMC9991433

[24] Fomotar M . Exploring the lived experience of male-to-female transgender youth accessing trans-related healthcare in los angeles (2016). University of San Diego. Available online at: https://digital.sandiego.edu/dissertations/43/ (Accessed May 15, 2025).

[25] Puckett JA Barr SM Wadsworth LP Thai J . Considerations for clinical work and research with transgender and gender diverse individuals. Behav Therapist. (2018) 41:253–262.

[26] Cohn TJ Casazza SP Cottrell EM . The mental health of gender and sexual minority groups in context. In Smalley KB Warren JC Barefoot KN (Eds.), LGBT health: Meeting the needs of gender and sexual minorities. Springer Publishing Company (2018), 161–179.

[27] To M Zhang Q Bradlyn A Getahun D Giammattei S Nash R . Visual conformity with affirmed gender or “Passing”: its distribution and association with depression and anxiety in a cohort of transgender people. J Sexual Med. (2020) 17:2084–92. doi: 10.1016/j.jsxm.2020.07.019, PMID: 32807706 PMC7529975

[28] Aldridge Z Patel S Guo B Nixon E Pierre Bouman W Witcomb GL . Long-term effect of gender-affirming hormone treatment on depression and anxiety symptoms in transgender people: A prospective cohort study. Andrology. (2021) 9:1808–16. doi: 10.1111/andr.12884, PMID: 32777129

[29] Colizzi M Costa R Todarello O . Transsexual patients’ psychiatric comorbidity and positive effect of cross-sex hormonal treatment on mental health: Results from a longitudinal study. Psychoneuroendocrinology. (2014) 39:65–73. doi: 10.1016/j.psyneuen.2013.09.029, PMID: 24275005

[30] Defreyne J T'Sjoen G Bouman WP Brewin N Arcelus J . Prospective evaluation of self-reported aggression in transgender persons. J Sexual Med. (2018) 15:768–76. doi: 10.1016/j.jsxm.2018.03.079, PMID: 29699761

[31] Fisher AD Castellini G Ristori J Casale H Cassioli E Sensi C . Cross-sex hormone treatment and psychobiological changes in transsexual persons: two-year follow-up data. J Clin Endocrinol Metab. (2016) 101:4260–9. doi: 10.1210/jc.2016-1276, PMID: 27700538

[32] Weinforth G Fakin R Giovanoli P Nuñez DG . Quality of life following male-to-female sex reassignment surgery. Deutsches Ärzteblatt Int. (2019) 116:253–260. doi: 10.3238/arztebl.2019.0253, PMID: 31130156 PMC6546862

[33] Wiepjes CM Nota NM De Blok CJM Klaver M De Vries ALC Wensing-Kruger SA . The amsterdam cohort of gender dysphoria study, (1972–2015): trends in prevalence, treatment, and regrets. J Sexual Med. (2018) 15:582–90. doi: 10.1016/j.jsxm.2018.01.016, PMID: 29463477

[34] Billings HM Boskey ER . Sexual health outcomes of non-facial gender-affirming surgery: a narrative review. Plast Aesthetic Res. (2025) 12. doi: 10.20517/2347-9264.2025.52

[35] Glynn TR Gamarel KE Kahler CW Iwamoto M Operario D Nemoto T . The role of gender affirmation in psychological well-being among transgender women. Psychol Sexual Orientation Gender Diversity. (2016) 3:336–44. doi: 10.1037/sgd0000171, PMID: 27747257 PMC5061456

[36] Papadopulos NA Lellé J-D Zavlin D Herschbach P Henrich G Kovacs L . Quality of life and patient satisfaction following male-to-female sex reassignment surgery. J Sexual Med. (2017) 14:721–30. doi: 10.1016/j.jsxm.2017.01.022, PMID: 28366591

[37] Van De Grift TC Elaut E Cerwenka SC Cohen-Kettenis PT De Cuypere G Richter-Appelt H . Effects of medical interventions on gender dysphoria and body image: A follow-up study. Psychosomatic Med. (2017) 79:815–23. doi: 10.1097/PSY.0000000000000465, PMID: 28319558 PMC5580378

[38] Shelemy L Cotton S Crane C Knight M . Systematic review of prospective adult mental health outcomes following affirmative interventions for gender dysphoria. Int J Transgender Health. (2025) 26:480–500. doi: 10.1080/26895269.2024.2333525, PMID: 40756726 PMC12312132

[39] Lindqvist EK Sigurjonsson H Möllermark C Rinder J Farnebo F Lundgren TK . Quality of life improves early after gender reassignment surgery in transgender women. Eur J Plast Surg. (2017) 40:223–6. doi: 10.1007/s00238-016-1252-0, PMID: 28603386 PMC5440516

[40] Valentine SE Shipherd JC . A systematic review of social stress and mental health among transgender and gender non-conforming people in the United States. Clin Psychol Rev. (2018) 66:24–38. doi: 10.1016/j.cpr.2018.03.003, PMID: 29627104 PMC6663089

[41] Wang J Mann F Lloyd-Evans B Ma R Johnson S . Associations between loneliness and perceived social support and outcomes of mental health problems: a systematic review. BMC Psychiatry. (2018) 18:156. doi: 10.1186/s12888-018-1736-5, PMID: 29843662 PMC5975705

[42] Gariépy G Honkaniemi H Quesnel-Vallée A . Social support and protection from depression: systematic review of current findings in Western countries. Br J Psychiatry. (2016) 209:284–93. doi: 10.1192/bjp.bp.115.169094, PMID: 27445355

[43] Boza C Nicholson Perry K . Gender-related victimization, perceived social support, and predictors of depression among transgender Australians. Int J Transgenderism. (2014) 15:35–52. doi: 10.1080/15532739.2014.890558

[44] Nemoto T Bödeker B Iwamoto M . Social support, exposure to violence and transphobia, and correlates of depression among male-to-female transgender women with a history of sex work. Am J Public Health. (2011) 101:1980–8. doi: 10.2105/AJPH.2010.197285, PMID: 21493940 PMC3222349

[45] Pflum SR Testa RJ Balsam KF Goldblum PB Bongar B . Social support, trans community connectedness, and mental health symptoms among transgender and gender nonconforming adults. Psychol Sexual Orientation Gender Diversity. (2015) 2:281–6. doi: 10.1037/sgd0000122

[46] Claes L Bouman WP Witcomb G Thurston M Fernandez-Aranda F Arcelus J . Non-suicidal self-injury in trans people: associations with psychological symptoms, victimization, interpersonal functioning, and perceived social support. J Sexual Med. (2015) 12:168–79. doi: 10.1111/jsm.12711, PMID: 25283073

[47] Bauer GR Scheim AI Pyne J Travers R Hammond R . Intervenable factors associated with suicide risk in transgender persons: a respondent driven sampling study in Ontario, Canada. BMC Public Health. (2015) 15:525. doi: 10.1186/s12889-015-1867-2, PMID: 26032733 PMC4450977

[48] Breidenstein A Hess J Hadaschik B Teufel M Tagay S . Psychosocial resources and quality of life in transgender women following gender-affirming surgery. J Sexual Med. (2019) 16:1672–80. doi: 10.1016/j.jsxm.2019.08.007, PMID: 31570138

[49] Kaptan S Cesur E Başar K Yüksel Ş . Gender dysphoria and perceived social support: A matched case-control study. J Sexual Med. (2021) 18:812–20. doi: 10.1016/j.jsxm.2021.01.174, PMID: 33573997

[50] Valente PK Dworkin JD Dolezal C Singh AA LeBlanc AJ Bockting WO . Prospective relationships between stigma, mental health, and resilience in a multi-city cohort of transgender and nonbinary individuals in the United States 2016–2019. Soc Psychiatry Psychiatr Epidemiol. (2022) 57:1445–56. doi: 10.1007/s00127-022-02270-6, PMID: 35312828

[51] Winter S Chalungsooth P Teh YK Rojanalert N Maneerat K Wong YW . Transpeople, transprejudice and pathologization: A seven-country factor analytic study. Int J Sexual Health. (2009) 21:96–118. doi: 10.1080/19317610902922537

[52] Yadegarfard M Meinhold-Bergmann ME Ho R . Family rejection, social isolation, and loneliness as predictors of negative health outcomes (Depression, suicidal ideation, and sexual risk behavior) among thai male-to-female transgender adolescents. J LGBT Youth. (2014) 11:347–63. doi: 10.1080/19361653.2014.910483

[53] Folkman S Lazarus RS . Coping as a mediator of emotion. J Pers Soc Psychol. (1988) 54:466–75. doi: 10.1037/0022-3514.54.3.466 3361419

[54] Grant DM Wingate LR Rasmussen KA Davidson CL Slish ML Rhoades-Kerswill S . An examination of the reciprocal relationship between avoidance coping and symptoms of anxiety and depression. J Soc Clin Psychol. (2013) 32:878–96. doi: 10.1521/jscp.2013.32.8.878

[55] Holahan CJ Moos RH Holahan CK Brennan PL Schutte KK . Stress generation, avoidance coping, and depressive symptoms: A 10-year model. J Consulting Clin Psychol. (2005) 73:658–66. doi: 10.1037/0022-006X.73.4.658, PMID: 16173853 PMC3035563

[56] Blalock JA Joiner TE . Interaction of cognitive avoidance coping and stress in predicting depression/anxiety. Cogn Ther Res. (2000) 24:47–65. doi: 10.1023/A:1005450908245

[57] Carvalho JP Hopko DR . Behavioral theory of depression: Reinforcement as a mediating variable between avoidance and depression. J Behav Ther Exp Psychiatry. (2011) 42:154–62. doi: 10.1016/j.jbtep.2010.10.001, PMID: 21315876

[58] Lindley L Bauerband L . The mediating role of avoidant and facilitative coping on the relation between discrimination and alcohol use among transgender and gender-diverse individuals. Transgender Health. (2023) 8:500–8. doi: 10.1089/trgh.2021.0173, PMID: 38130979 PMC10732159

[59] White Hughto JM Pachankis JE Willie TC Reisner SL . Victimization and depressive symptomology in transgender adults: The mediating role of avoidant coping. J Couns Psychol. (2017) 64:41–51. doi: 10.1037/cou0000184, PMID: 28068130 PMC5226079

[60] Freese R Ott MQ Rood BA Reisner SL Pantalone DW . Distinct coping profiles are associated with mental health differences in transgender and gender nonconforming adults. J Clin Psychol. (2018) 74:136–46. doi: 10.1002/jclp.22490, PMID: 28608524

[61] Sin LYN . Psychological adjustment of transgender individuals in Hong Kong: the roles of gender identity acceptance, social support and coping styles. HKU Theses Online (HKUTO). (2022).

[62] Lovibond SH . Manual for the depression anxiety stress scales. Sydney Psychol foundation. (1995). doi: 10.1037/t01004-000

[63] Moussa MT Lovibond PF Laube R . (2001). Psychometric properties of a Chinese version of the 21-item depression anxiety stress scales (DASS21). Sydney, NSW: Transcultural Mental Health Centre. Cumberland Hospital.

[64] Jones BA Haycraft E Murjan S Arcelus J . Body dissatisfaction and disordered eating in trans people: A systematic review of the literature. Int Rev Psychiatry. (2016) 28:81–94. doi: 10.3109/09540261.2015.1089217, PMID: 26618239

[65] Zimet GD Dahlem NW Zimet SG Farley GK . The multidimensional scale of perceived social support. J Pers Assess. (1988) 52:30–41. doi: 10.1207/s15327752jpa5201_2, PMID: 2280326

[66] Chou K-L . Assessing Chinese adolescents’ social support: the multidimensional scale of perceived social support. Pers Individ Dif. (2000) 28:299–307. doi: 10.1016/S0191-8869(99)00098-7

[67] Carver CS . You want to measure coping but your protocol’ too long: Consider the brief cope. Int J Behav Med. (1997) 4:92–100. doi: 10.1207/s15327558ijbm0401_6, PMID: 16250744

[68] Carver CS Scheier MF Weintraub JK . Assessing coping strategies: A theoretically based approach. J Pers Soc Psychol. (1989) 56:267–83. doi: 10.1037/0022-3514.56.2.267 2926629

[69] Tang KN Chan CS Ng J Yip C-H . Action type-based factorial structure of Brief COPE among Hong Kong Chinese. J Psychopathol Behav Assess. (2016) 38:631–44. doi: 10.1007/s10862-016-9551-0

[70] Hembree WC Cohen-Kettenis P Delemarre-van De Waal HA Gooren LJ Meyer WJ Spack NP . Endocrine treatment of transsexual persons : an endocrine society clinical practice guideline. J Clin Endocrinol Metab. (2009) 94:3132–54. doi: 10.1210/jc.2009-0345, PMID: 19509099

[71] Alzahrani T Nguyen T Ryan A Dwairy A McCaffrey J Yunus R . Cardiovascular disease risk factors and myocardial infarction in the transgender population. Circulation: Cardiovasc Qual Outcomes. (2019) 12:e005597. doi: 10.1161/CIRCOUTCOMES.119.005597, PMID: 30950651

[72] Dhejne C Lichtenstein P Boman M Johansson ALV Långström N Landén M . Long-term follow-up of transsexual persons undergoing sex reassignment surgery: cohort study in Sweden. PloS One. (2011) 6:e16885. doi: 10.1371/journal.pone.0016885, PMID: 21364939 PMC3043071

[73] Marshall E Claes L Bouman WP Witcomb GL Arcelus J . Non-suicidal self-injury and suicidality in trans people: A systematic review of the literature. Int Rev Psychiatry. (2016) 28:58–69. doi: 10.3109/09540261.2015.1073143, PMID: 26329283

[74] Ghabrial MA Scheim AI Chih C Santos H Adams NJ Bauer GR . Change in finances, peer access, and mental health among trans and nonbinary people during the COVID-19 pandemic. LGBT Health. (2023) 10:595–607. doi: 10.1089/lgbt.2022.0296, PMID: 37347954 PMC10712362

[75] Dhejne C Van Vlerken R Heylens G Arcelus J . Mental health and gender dysphoria: A review of the literature. Int Rev Psychiatry. (2016) 28:44–57. doi: 10.3109/09540261.2015.1115753, PMID: 26835611

[76] Marshall E Claes L Bouman WP Witcomb GL Arcelus J . Non-suicidal self-injury and suicidality in trans people: A systematic review of the literature. Gender Dysphoria Gender Incongruence. (2018) 28:70–81., PMID: 26329283 10.3109/09540261.2015.1073143

[77] Trew JL . Exploring the roles of approach and avoidance in depression: An integrative model. Clin Psychol Rev. (2011) 31:1156–68. doi: 10.1016/j.cpr.2011.07.007, PMID: 21855826

[78] Aderka IM McLean CP Huppert JD Davidson JRT Foa EB . Fear, avoidance and physiological symptoms during cognitive-behavioral therapy for social anxiety disorder. Behav Res Ther. (2013) 51:352–8. doi: 10.1016/j.brat.2013.03.007, PMID: 23639301 PMC3669252

[79] Budge SL Adelson JL Howard KAS . Anxiety and depression in transgender individuals: The roles of transition status, loss, social support, and coping. J Consulting Clin Psychol. (2013) 81:545–57. doi: 10.1037/a0031774, PMID: 23398495

[80] Veale JF Peter T Travers R Saewyc EM . Enacted stigma, mental health, and protective factors among transgender youth in Canada. Transgender Health. (2017) 2:207–16. doi: 10.1089/trgh.2017.0031, PMID: 29279875 PMC5734137

[81] Alanko K Lund H . Transgender youth and social support: A survey study on the effects of good relationships on well-being and mental health. YOUNG. (2020) 28:199–216. doi: 10.1177/1103308819850039

[82] Dowers E White C Cook K Kingsley J . Trans, gender diverse and non-binary adult experiences of social support: A systematic quantitative literature review. Int J Transgender Health. (2020) 21:242–57. doi: 10.1080/26895269.2020.1771805, PMID: 34993509 PMC8726637

[83] Scandurra C Amodeo AL Valerio P Bochicchio V Frost DM . Minority stress, resilience, and mental health: A study of Italian transgender people. J Soc Issues. (2017) 73:563–85. doi: 10.1111/josi.12232

