# Supplementary material for: Effect of gender-affirming treatments on depression and anxiety symptoms in transgender people: a retrospective cohort study
Source: Front Psychiatry. 2026 Jan 5;16:1709778. doi: 10.3389/fpsyt.2025.1709778 (PMC12812681; doi:10.3389/fpsyt.2025.1709778)
Supplement: Supplementary file 1 [file Table1.docx]

Supplementary Material

Supplementary Table 1. Sensitivity analysis of updated GAHT duration (excluding OTC period)

|  | **Depression** | |  | **Anxiety** | |  | **Stress** | |
| --- | --- | --- | --- | --- | --- | --- | --- | --- |
| **Variable** | **Estimate (95% CI)** | **p-value** |  | **Estimate (95% CI)** | **p-value** |  | **Estimate (95% CI)** | **p-value** |
| **Time [1]** | 0.950 (0.703 –  1.284) | 0.741 |  | 0.920 (0.698 –  1.213) | 0.557 |  | 0.986 (0.727 –  1.338) | 0.929 |
| **Group [GAS During Cohort]** | 1.107 (0.749 –  1.635) | 0.611 |  | 0.988 (0.675 –  1.447) | 0.952 |  | 1.050 (0.728 –  1.514) | 0.793 |
| **GAHT Duration before Year 5** | 1.059 (0.886 –  1.266) | 0.530 |  | 1.019 (0.852 –  1.218) | 0.839 |  | 1.048 (0.893 –  1.230) | 0.568 |
| **GAHT Duration after Year 5** | 0.985 (0.944 –  1.028) | 0.483 |  | 0.970 (0.929 –  1.012) | 0.164 |  | 0.983 (0.946 –  1.021) | 0.366 |
| **Family Support** | 0.978 (0.963 –  0.994) | ***0.007** |  | 0.989 (0.975 –  1.004) | 0.166 |  | 0.988 (0.973 –  1.003) | 0.107 |
| **Friends and Significant Others Support** | 0.987 (0.979 –  0.995) | ***0.003** |  | 0.992 (0.984 –  1.000) | ***0.049** |  | 0.992 (0.984 –  1.000) | ***0.043** |
| **Facilitative Coping** | 0.959 (0.934 –  0.984) | ***0.002** |  | 0.962 (0.938 –  0.986) | ***0.003** |  | 0.985 (0.961 –  1.010) | 0.238 |
| **Avoidant Coping** | 1.107 (1.071 –  1.144) | ***<0.001** |  | 1.091 (1.057 –  1.126) | ***<0.001** |  | 1.106 (1.072 –  1.141) | ***<0.001** |
| **Time [1] x Group [GAS During Cohort]** | 1.086 (0.725 –  1.626) | 0.691 |  | 1.080 (0.748 –  1.559) | 0.682 |  | 1.140 (0.754 –  1.725) | 0.536 |
|  | Random Intercept Variance = 0.33; Residual Variance = 0.46 | |  | Random Intercept Variance = 0.39; Residual Variance = 0.37 | |  | Random Intercept Variance = 0.19; Residual Variance = 0.50 | |
| *GAS, gender-affirming surgery; GAHT, gender-affirming hormone therapy; OTC, over-the-counter; *p<0.05* | | | | | | | | |

Supplementary Table 2. Sensitivity analysis of transition status (FtM, MtF, NB)

|  | **Depression** | |  | **Anxiety** | |  | **Stress** | |
| --- | --- | --- | --- | --- | --- | --- | --- | --- |
| **Variable** | **Estimate (95% CI)** | **p-value** |  | **Estimate (95% CI)** | **p-value** |  | **Estimate (95% CI)** | **p-value** |
| **Time [1]** | -4.362 (-12.703 –  3.980) | 0.314 |  | -4.420 (-10.943 –  2.103) | 0.195 |  | -3.132 (-11.552 –  5.287) | 0.472 |
| **Transition Status [MtF]** | -6.508 (-15.030 –  2.014) | 0.140 |  | -0.304 ( -7.520 –  6.912) | 0.934 |  | -4.065 (-12.449 –  4.319) | 0.346 |
| **Transition Status [NB]** | -9.359 (-23.039 –  4.321) | 0.185 |  | -4.939 (-16.674 –  6.796) | 0.413 |  | -12.125 (-25.531 –  1.281) | 0.082 |
| **GAHT Duration by Year 2** | -2.088 ( -5.447 –  1.271) | 0.233 |  | -1.587 ( -4.250 –  1.076) | 0.253 |  | -0.347 ( -3.718 –  3.025) | 0.842 |
| **GAHT Duration Year 2-5** | 0.460 ( -1.655 –  2.574) | 0.673 |  | 1.004 ( -0.662 –  2.670) | 0.247 |  | -0.584 ( -2.709 –  1.540) | 0.594 |
| **GAHT Duration after Year 5** | -0.385 ( -0.986 –  0.217) | 0.220 |  | -0.016 ( -0.535 –  0.504) | 0.953 |  | -0.021 ( -0.608 –  0.567) | 0.945 |
| **Family Support** | -0.245 ( -0.529 –  0.040) | 0.103 |  | -0.254 ( -0.484 – -0.025) | ***0.039** |  | -0.120 ( -0.404 –  0.164) | 0.413 |
| **Friends and Significant Others Support** | -0.096 ( -0.246 –  0.055) | 0.222 |  | -0.051 ( -0.173 –  0.071) | 0.421 |  | -0.119 ( -0.269 –  0.030) | 0.128 |
| **Facilitative Coping** | -0.653 ( -1.186 – -0.120) | ***0.023** |  | -0.454 ( -0.890 – -0.018) | 0.051 |  | -0.460 ( -0.988 –  0.067) | 0.098 |
| **Avoidant Coping** | 1.176 (  0.592 –  1.761) | ***<0.001** |  | 0.675 (  0.196 –  1.154) | ***0.01** |  | 1.117 (  0.537 –  1.696) | ***<0.001** |
| **Time [1] x Transition Status [MtF]** | 6.234 ( -1.683 – 14.152) | 0.134 |  | 3.074 ( -3.002 –  9.149) | 0.330 |  | 4.006 ( -4.031 – 12.042) | 0.337 |
| **Time [1] x Transition Status [NB]** | 21.396 (  7.935 – 34.856) | ***0.004** |  | 8.647 ( -1.614 – 18.909) | 0.110 |  | 15.298 (  1.609 – 28.987) | **0.037** |
|  | Random Intercept Variance = 38.5; Residual Variance = 39.8 | |  | Random Intercept Variance = 36.1; Residual Variance = 22.4 | |  | Random Intercept Variance = 33.3; Residual Variance = 41.5 | |
| *FtM, female-to-male; MtF, male-to-female; NB, non-binary; GAHT, gender-affirming hormone therapy; *p<0.05* | | | | | | | | |

Supplementary Table 3. Sensitivity analysis of above-threshold DASS-21 scores

|  | **Depression** | |  | **Anxiety** | |  | **Stress** | |
| --- | --- | --- | --- | --- | --- | --- | --- | --- |
| **Variable** | **Estimate (95% CI)** | **p-value** |  | **Estimate (95% CI)** | **p-value** |  | **Estimate (95% CI)** | **p-value** |
| **Time [1]** | -3.102 ( -8.087 –  1.882) | 0.228 |  | -1.265 (-5.590 –  3.060) | 0.569 |  | 2.101 ( -3.680 –  7.882) | 0.480 |
| **Group [GAS During Cohort]** | 2.447 ( -2.351 –  7.244) | 0.321 |  | -0.503 (-4.335 –  3.329) | 0.798 |  | 0.590 ( -4.545 –  5.725) | 0.823 |
| **GAHT Duration by Year 2** | -1.075 ( -3.768 –  1.618) | 0.438 |  | 0.010 (-2.286 –  2.307) | 0.993 |  | -0.205 ( -3.117 –  2.708) | 0.891 |
| **GAHT Duration Year 2-5** | 1.248 ( -0.688 –  3.185) | 0.212 |  | -0.507 (-2.071 –  1.056) | 0.528 |  | -1.564 ( -3.558 –  0.430) | 0.131 |
| **GAHT Duration after Year 5** | -0.379 ( -0.873 –  0.114) | 0.138 |  | -0.189 (-0.569 –  0.191) | 0.334 |  | -0.274 ( -0.774 –  0.226) | 0.288 |
| **Family Support** | -0.232 ( -0.453 – -0.011) | ***0.045** |  | -0.126 (-0.318 –  0.066) | 0.203 |  | -0.128 ( -0.388 –  0.131) | 0.337 |
| **Friends and Significant Others Support** | -0.103 ( -0.213 –  0.006) | 0.070 |  | -0.020 (-0.117 –  0.078) | 0.691 |  | -0.103 ( -0.235 –  0.029) | 0.132 |
| **Facilitative Coping** | -0.490 ( -0.862 – -0.117) | ***0.013** |  | -0.250 (-0.537 –  0.036) | 0.092 |  | 0.007 ( -0.384 –  0.398) | 0.973 |
| **Avoidant Coping** | 1.077 (  0.598 –  1.555) | ***<0.001** |  | 0.697 ( 0.302 –  1.091) | ***0.001** |  | 0.973 (  0.456 –  1.491) | ***<0.001** |
| **Time [1] x Group [GAS During Cohort]** | -5.736 (-11.392 – -0.079) | 0.052 |  | -2.284 (-7.031 –  2.464) | 0.350 |  | -1.368 ( -7.563 –  4.827) | 0.667 |
|  | Random Intercept Variance = 17.7; Residual Variance = 40.4 | |  | Random Intercept Variance = 8.6; Residual Variance = 35.1 | |  | Random Intercept Variance = 13.5; Residual Variance = 51.0 | |
| *GAS, gender-affirming surgery; GAHT, gender-affirming hormone therapy; *p<0.05* | | | | | | | | |
